# Polyphest: fast polyploid phylogeny estimation

**DOI:** 10.1093/bioinformatics/btae390

**Published:** 2024-09-04

**Authors:** Zhi Yan, Zhen Cao, Luay Nakhleh

**Affiliations:** Department of Computer Science, Rice University, Houston, TX 77005, United States; Department of Computer Science, Rice University, Houston, TX 77005, United States; Department of Computer Science, Rice University, Houston, TX 77005, United States; Department of BioSciences, Rice University, Houston, TX 77005, United States

## Abstract

**Motivation:**

Despite the widespread occurrence of polyploids across the Tree of Life, especially in the plant kingdom, very few computational methods have been developed to handle the specific complexities introduced by polyploids in phylogeny estimation. Furthermore, methods that are designed to account for polyploidy often disregard incomplete lineage sorting (ILS), a major source of heterogeneous gene histories, or are computationally very demanding. Therefore, there is a great need for efficient and robust methods to accurately reconstruct polyploid phylogenies.

**Results:**

We introduce Polyphest (POLYploid PHylogeny ESTimation), a new method for efficiently and accurately inferring species phylogenies in the presence of both polyploidy and ILS. Polyphest bypasses the need for extensive network space searches by first generating a multilabeled tree based on gene trees, which is then converted into a (uniquely labeled) species phylogeny. We compare the performance of Polyphest to that of two polyploid phylogeny estimation methods, one of which does not account for ILS, namely PADRE, and another that accounts for ILS, namely MPAllopp. Polyphest is more accurate than PADRE and achieves comparable accuracy to MPAllopp, while being significantly faster. We also demonstrate the application of Polyphest to empirical data from the hexaploid bread wheat and confirm the allopolyploid origin of bread wheat along with the closest relatives for each of its subgenomes.

**Availability and implementation:**

Polyphest is available at https://github.com/NakhlehLab/Polyphest.

## 1 Introduction

Ploidy of an organism’s cell is the number of whole sets of chromosomes (i.e. genome) in the cell. The ploidy could be one (haploid cells) or larger (diploid, triploid, etc.). Polyploidization, also known as whole-genome duplication (WGD), is an increase (e.g. doubling) in the number of copies of the entire genome of a species. This increase could be the result of WGD or hybridization. In autopolyploidy, the number of chromosome copies is doubled. In allopolyploidy, two species hybridize and the hybrid offspring receives both chromosome sets from the parents.

Polyploidy is a crucial factor in speciation and genomic and phenotypic novelties (Oxelman *et al.* 2017, Blischak *et al.* 2018), particularly in plants (Heslop-Harrison *et al.* 2023). Given the evolutionary role of polyploidy in both wild and cultivated plants, it has been used as a tool for improving plant vigor by plant breeders (Sattler *et al.* 2016). Recent research also suggests polyploidy’s importance in animal evolution. The identification of two ancient WGD events at the base of the vertebrate lineage, known as 2R-WGD events (Dehal and Boore 2005), highlights its potential contribution to the radiation of vertebrates. These ancestral genome duplications are believed to be responsible for the emergence of key gene families linked to development (e.g. Hox genes) and the immune system (Nei *et al.* 1997, Holland 1999).

Species phylogeny estimation in the presence of polyploids is very challenging. The wide array of recently introduced species tree inference methods (Mirarab *et al.* 2021) primarily relies on orthologous genes, but polyploids present duplicated genes that might be paralogs (duplicates within a species) or homoeologs (duplicates from separate parental species). This complexity often leads to the exclusion of polyploids from phylogenetic studies. Recent advances offer promising avenues for unraveling these intricate relationships. Phylogenetic networks (Elworth *et al.* 2019), a more complex model than trees, allow for modeling non-treelike evolution often seen in polyploidy. However, as discussed by Yan *et al.* (2021), most phylogenetic network inference methods fail to deal adequately with polyploidy. Multilabeled trees, or MUL-trees (Huber *et al.* 2006), are yet another mathematical model that allows for capturing polyploids. They extend traditional phylogenetic trees by allowing multiple leaves to be labeled by the same taxon name in order to capture multiple copies of the same subgenome that arise due to polyploidy. There is a close relationship between phylogenetic networks and MUL-trees that can be analyzed through “folding” and “unfolding” operations (Huber *et al.* 2016), which, in turn, provides a framework for disentangling the evolutionary history of polyploids (Huber and Maher 2023).

A number of phylogenetic methods leverage MUL-trees for analyzing polyploidy. PADRE (Lott *et al.* 2009), for example, adapts the greedy consensus strategy to generate a MUL-tree, which is subsequently converted into a network via the algorithm of Huber *et al.* (2006). GRAMPA (Thomas *et al.* 2017), on the other hand, builds upon a parsimonious reconciliation algorithm to identify polyploidization events and their modes within a MUL-tree framework. However, these methods do not account for incomplete lineage sorting (ILS), a pervasive evolutionary process that can cause discordance among gene trees (Mirarab *et al.* 2021). While polyploid phylogeny estimation methods such as AlloppNET (Jones *et al.* 2013, Jones 2017), a fully parametric Bayesian approach that jointly estimates the gene trees and species phylogeny, and MPAllopp (Yan *et al.* (2021)), a parsimony method that infers the species network by minimizing deep coalescences, account for ILS, they have their limitations, including a restricted set of polyploid types, computational requirements, scalability issues, and the prerequisite of prior knowledge regarding subgenome assignments, which may not always be available or accurate.

To address these limitations, we introduce Polyphest (POLYploid PHylogeny ESTimation), a method that builds on the PADRE algorithm (Huber *et al.* 2006, Lott *et al.* 2009) to accommodate ILS. Polyphest employs a multistep approach. It first decomposes the input multilabeled gene trees into a collection of clusters and constructs an approximate compatibility graph. By solving the maximum-weight clique problem on this graph, Polyphest efficiently selects highly supported clusters. These selected clusters are then used to build a greedy consensus MUL-tree. Finally, Polyphest folds the MUL-tree into a (uniquely labeled) phylogenetic network by merging near-isomorphic subtrees.

We assess in simulations the accuracy and efficiency of Polyphest as compared to both PADRE and MPAllopp and demonstrate its applicability to biological data. To the best of our knowledge, Polyphest is the first method that simultaneously accounts for polyploidy and ILS (a limitation of PADRE, since it does not account for ILS) while directly estimating phylogenetic networks from summarized gene trees without requiring exhaustive exploration of the network space (a limitation of MPAllopp that traverses the network space explicitly).

## 2 Background

**Figure 1.**
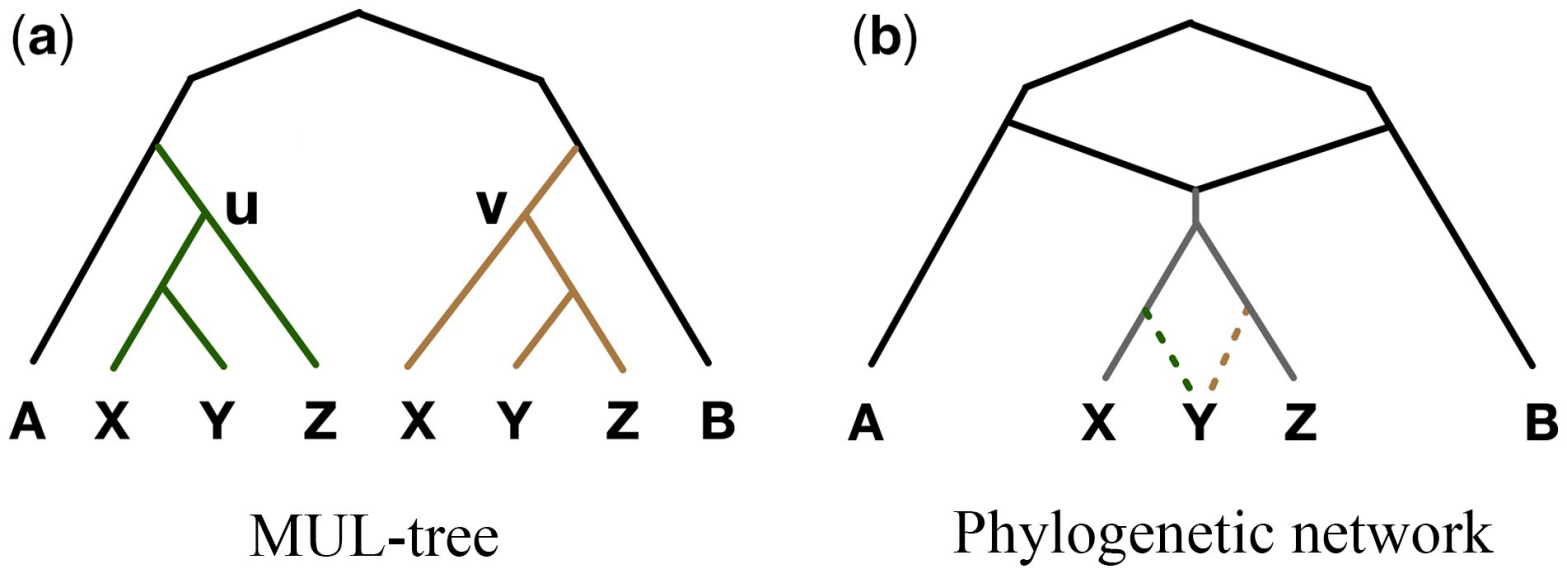
Illustration of MUL-trees and phylogenetic networks. The leaves of a MUL-tree are not uniquely labeled. The leaves of a phylogenetic network are uniquely labeled, but it has nodes of in-degree 2

A *phylogenetic network* on set X of taxa is a rooted, directed, acyclic graph Ψ=(V,E) with $V(\Psi )=\{V_{L},V_{T},V_{R}\}$, where, *V_L_* is the set of leaf nodes (out-degree of 0), *V_T_* is the set of internal tree nodes (in-degree of 1, except for the root node *ρ*, which has an in-degree of 0, and out-degree ≥2), and *V_R_* is the set of internal reticulation nodes (in-degree of 2 and out-degree of 1). Every node in the network is reachable from the root *ρ*. E(Ψ) is the set of Ψ’s directed edges. If $V_{R}=\emptyset$, then Ψ is a rooted phylogenetic tree. A node whose out-degree is >2 is called *unresolved* or *multifurcating*. In a phylogenetic tree or network, the nodes in *V_L_* are uniquely labeled by elements of X. The leaves of Ψ are labeled, $\phi :V_{L}\to X$. For tree Ψ, if ϕ is injective, then Ψ is a (uniquely labeled) phylogenetic tree. If ϕ is not injective, then Ψ is a multilabeled tree, or MUL-tree. Figure 1 shows a MUL-tree and a phylogenetic network. Hereafter, “tree” and “phylogenetic tree” refer to uniquely labeled trees; otherwise, we use “MUL-tree.” Furthermore, when the phylogeny is a tree, we typically use *T* instead of Ψ to denote the phylogeny.

For any node *v* in tree *T*, we denote by *T_v_* the *clade* or subtree of *T* that is rooted at *v* and by $C_{T}(v)$ the *cluster* of labels of *T_v’_*s leaves. The set of clusters induced by a tree *T* is $C(T)=\cup_{v\in V(T)}\{C_{T}(v)\}$. In trees, clusters are represented as sets, while in MUL-trees they are represented as multisets. Clusters induced by the root or leaves are referred to as *trivial* clusters, while others are called *non-trivial* clusters. Two clusters *C*(*u*) and *C*(*v*), where each cluster consists of unique elements, are said to be *compatible* if one of the following conditions holds: C(u)⊆C(v), C(v)⊆C(u), or C(u)∩C(v)=∅. A cluster is said to be *compatible* with a tree *T* if it is compatible with every cluster induced by *T*. Given a collection C of pairwise compatible clusters on X, there exists a unique rooted tree that induces C (Semple *et al.* 2003).

## 3 Materials and methods

### 3.1 MUL-tree construction

#### 3.1.1 Greedy consensus MUL-tree and PADRE

We begin by revisiting the greedy consensus MUL-tree problem, as detailed by Lott *et al.* (2009). Let $T=\{T_{1},\ldots ,T_{n}\}$ be a collection of rooted trees leaf-labeled by set X and $C(T)=\cup_{i=1}^{n}C(T_{i})$. Given the nature of MUL-trees, it is possible for a cluster to occur more than once; $m_{T}(C)$ denotes the multiplicity of cluster *C* in MUL-tree *T*. We represent C(T) as a set of cluster-multiplicity pairs: $\left\{(C_{T}^{1},m_{T}(C_{T}^{1})),\ldots ,(C_{T}^{k},m_{T}(C_{T}^{k}))\right\}$. For simplicity, we write the cluster-multiplicity pair as $\tilde{C}=(C,m)$. For example, for the MUL-tree in Fig. 1a, cluster {*x*, *y*, *z*} appears twice ($C_{T}(u)$ and $C_{T}(v)$) in this MUL-tree, so it has a multiplicity of 2, denoted as $\tilde{C}=(\{x,y,z\},2)$.

Extending this concept to the entire collection T, we view the induced clusters as a list of cluster-multiplicity pairs $\left[{\tilde{C}}^{i,j}=(C^{i},m^{i,j})\right]$ sorted in descending order by their frequencies $s_{T}({\tilde{C}}^{i,j})=|\{T_{k}|{\tilde{C}}^{i,j}\in C(T_{k})\}|$. It is crucial to distinguish between multiplicity and frequency. Multiplicity refers to how many times a specific cluster appears within a single MUL-tree. Frequency, on the other hand, considers how often a cluster appears with a particular multiplicity across multiple trees. For example, cluster {*x*, *y*, *z*} might appear twice (multiplicity of 2) in one tree and only once (multiplicity of 1) in another. Its frequency for a multiplicity of 2 then depends on how many other trees share that specific repetition level (i.e. 2), for {*x*, *y*, *z*}.

We define function ComputeSortedClusters to compute the clusters induced by T computing the clusters and their corresponding multiplicities within each individual tree. As it iterates through the trees, it also calculates the frequency of each cluster-multiplicity pair across the entire collection. The output of ComputeSortedClusters is a list of cluster-multiplicity pairs induced by T (denoted as $C_{T}$), along with their corresponding frequencies (denoted as $s_{T}$) that is sorted in descending order by frequency.

The greedy consensus MUL-tree is built by iteratively adding clusters from this sorted list to an initially empty set. A cluster is added only if it is compatible with those already in the set so that the resulting set of clusters defines a tree.

PADRE (Lott *et al.* (2009)) adopts the greedy strategy but with some key modifications. First, it filters out clusters whose multiplicity is smaller than a predefined threshold. Then, it partitions C(T) into “core” and “ambiguous” clusters. Core clusters contain at least one label with multiplicity 1 across all clusters in C(T). These labels can be thought of as single copies of diploids. Both core and ambiguous clusters are then sorted by their multiplicities within the input trees. PADRE prioritizes core clusters during the iterative addition process to construct a backbone tree, then integrates ambiguous clusters one by one to further refine the consensus MUL-tree. Full details of the method can be found in Lott *et al.* (2009).

#### 3.1.2 Reducing the number of clusters in Polyphest

In contrast to PADRE, Polyphest first reduces the number of candidate clusters. While straightforward for trees, cluster reduction becomes challenging for MUL-trees. This difficulty arises because the compatibility rule used for trees no longer holds for MUL-trees. For instance, consider MUL-tree *T* with root *w* that has two children *u* and *v*, and *u* has two leaf children labeled by *x* and *z* and *v* has three leaf children labeled by *x*, *y*, and *z*. The cluster {*x*, *y*} would be regarded as incompatible with $C_{T}(u)$ under the tree compatibility definition, making it seem incompatible with *T*. Determining true compatibility for a collection of clusters on a multiset is NP-hard (Huber *et al.* 2008). To address this complexity, PADRE forgoes cluster reduction and assigns priorities to core and ambiguous clusters. Polyphest, on the other hand, tackles this issue by introducing the notion of *approximate compatibility*.

Definition 1. (Approximate Compatibility) Two clusters with multiplicities (*C*_1_, *m*_1_) and (*C*_2_, *m*_2_), drawn from the multiset L of leaf labels of a MUL-tree, are considered approximately compatible if any of these conditions hold: $C_{1}\subseteq C_{2},\;C_{1}⊇C_{2}$, or $L\setminus \cup_{i=1}^{m_{1}}C_{1}⊇\cup_{i=1}^{m_{2}}C_{2}$, where the union is that of multisets.

Polyphest makes use of maximum-weight approximately compatible clusters.

Problem 1. (Maximum Weight Approximate Compatible Clusters (MWACC)) Let $T=\{T_{1},\ldots ,T_{n}\}$ be a collection of trees on a taxon set X. Let L be a target multiset such that for each element l∈L, l∈X. Additionally, let *w* be a weight function that assigns a weight to each potential cluster. The output is a collection C of clusters, where each cluster is a submultiset of L and originates from the clusters induced by the collection T. Furthermore, all clusters in C must be pairwise approximately compatible. The objective is to maximize the total weight of the clusters in C as given by $W(C)=\sum_{\tilde{C}\in C}w(\tilde{C})$.

Unlike PADRE, which prioritizes core clusters, Polyphest utilizes all clusters due to the observation that ILS could weaken the importance of core clusters. Specifically, including a misleading core cluster that is incompatible with the true MUL-tree could even block the inclusion of other informative, highly supported ambiguous clusters.

***Approximate compatibility graph construction***. We leverage the sorted list of clusters with frequencies, generated by ComputeSortedClusters, and the concept of approximate compatibility to construct an approximate compatibility graph *G_AC_*. Each node in *G_AC_* represents a cluster ${\tilde{C}}^{i,j}$ in C(T). Two nodes are connected by an edge if their corresponding clusters are approximately compatible. Node *v* representing cluster ${\tilde{C}}^{i,j}$ is assigned weight $w(v)=m^{i,j}\cdot s({\tilde{C}}^{i,j})\cdot |C^{i}{|}^{0.65}$. This weighting scheme reflects several considerations. Given the heterogeneity introduced by ILS, the true MUL-tree might contain multiple copies of a cluster like ${\tilde{C}}^{i,j}$ where *j *>* *1. However, a cluster ${\tilde{C}}^{i,k}$ where *k *<* j* may be more prevalent across T, while ${\tilde{C}}^{i,j}$ hold more valuable information. Therefore, we use the term $m^{i,j}\cdot s({\tilde{C}}^{i,j})$ to emphasize the importance of ${\tilde{C}}^{i,j}$. Furthermore, while smaller clusters are informative for refining unresolved nodes, their reliability is compromised by ILS. The exponent 0.65, chosen empirically, balances the impact of cluster size and the other factors (multiplicity and frequency). Polyphest identifies a clique in *G_AC_* with the maximum total weight, which represents an optimal collection of approximately compatible clusters.

***An integer linear programming solution***. We now present an integer linear programming (ILP) solution to the MWACC problem, denoted by the function SolveMWACC. Let $X=[x_{1},\ldots ,x_{n}]$ be a binary vector where each binary variable *x_k_* reflects the inclusion status of cluster ${\tilde{C}}^{i,j}$. The function f:{1,…,n}→C(T) maps each selection variable *x_k_* to its corresponding cluster ${\tilde{C}}^{i,j}$. Additionally, we denote a approximate compatibility matrix as Λ, where $\Lambda_{i,j}=1$ if *f*(*i*) and *f*(*j*) are approximately compatible, and $\Lambda_{i,j}=0$ otherwise. The ILP model is as follows:

(1) $$\max\sum_{k=1}^{n}w_{k}\cdot x_{k}$$

subject to

(2) $$\sum_{k:f(k)=(C^{i},m^{i,j})\;\text{for any}\;j}x_{k}\leq 1\;\;\forall C^{i}\in C(T)$$

 

(3) $$x_{i}+x_{j}\leq 1\;\;\text{if}\;\Lambda_{i,j}=0$$

Constraints (2) and (3) stipulate that no more than one multiplicity of any cluster *C^i^* is selected and to prevent the selection of clusters that are not approximately compatible, respectively.

Algorithm 1. MUL-tree reconstruction algorithm

**Input:** A collection $G=\{g_{1},\ldots g_{n}\}$ of multilabeled gene trees on the taxon set X and multiset L of leaf labels.

**Output:** A consensus MUL-tree T on multiset L and cluster frequency map $s_{G}$.

1: $C(G),s_{G}\leftarrow$  ComputeSortedClusters(G);

2: $C_{AC}\leftarrow$  SolveMWACC(C(G),L);

3: Create a star tree *T* whose leaves are labeled by the elements of L;

4: **for all**  $C\in C_{AC}$  **do**

5:   **if** *C* is compatible with *T* **then**

6:    Add *C* to *T*;

7: **for all**  v∈V(T)  **do**

8:   **if** *v* is multifurcating **then**

9:    Refine(v,C(G));

 **return**  $T,s_{G}$;

Polyphest then uses the output (sorted) clusters obtained from the ILP solution (SolveMWACC) to construct a greedy consensus MUL-tree (Algorithm 1). Notice that this tree may not be strictly binary. To address this, we define function Refine, which operates on each multifurcating node in the tree. For each such node, Refine selects a binary refinement that maximizes the total weight (as defined in the ILP model) of the clusters induced by its children.

Algorithm 2. Network Construction Algorithm

**Input:** MUL-tree *T*, near-isomorphic threshold *δ*, cluster frequency map $s_{T}$

**Output:** MUL-tree *T* is modified in place to represent the network

1: Assign unique isomorphism codes to all nodes in *T*

2: L←list of H+1 empty queues ▹ *H* is the root height

3: L[H].enqueue(root node)

4: **for** *h *=* H* down to 0 **do**

5:  D←L[h] ▹ queue containing nodes of height *h*

6:  **while** not *D*.isEmpty() **do**

7:   u←D.popLeft()

8:   $\tilde{T}\leftarrow \;$  FindNearIsomorphicClades(u,D,δ)

9:   **if** not $\tilde{T}$.isEmpty() **then**

10:    MergeNearIsomorphicClades($\tilde{T},s_{T}$)

11:    Update *T* accordingly

12:   **for all** child node of *u* **do**

13:    Add the child to L[child.height]

14: **function**  FindNearIsomorphicClades(u,D,δ)

15:  $\tilde{T}\leftarrow$ an empty list

16:  **for all**  v∈D and v≠u  **do**

17:   **if**  AreNearIsomorphic(u,v,δ) **then**

18:    **if**  $\tilde{T}$.isEmpty() **then** Add *u* to $\tilde{T}$

19:    Add *v* to $\tilde{T}$

20:    Remove *v* from *D*

 **return**  $\tilde{T}$

21: **function**  AreNearIsomorphic(u,v,δ)

22:  **if** *u* and *v* do not have the same cluster **then return** False

23:  **if** *u*.code = *v*.code **then** ▹ Exact topology match

     **return** True

24:  d←  ComputeNormalizedGraphEditDistance(*u*, *v*)

         **return**  d≤δ ▹ Within near-isomorphism threshold

25: **function**  MergeNearIsomorphicClades($\tilde{T},s_{T}$)

26:  maxSupport ←0

27:  hybrid ← null

28:  **for all**  $u\in \tilde{T}$  **do**

29:   support ← ComputeCladeScore($u,s_{T}$)

30:   **if** support ≥ maxSupport **then**

31:    maxSupport ← support

32:    hybrid ←u

33:  Perform subdivision, identification, and pruning on $\tilde{T}\setminus \{$hybrid}

34: **function**  ComputeCladeScore($u,s_{T}$)

35:  Compute the collection C of clusters induced by clade *u*

36:  totalScore ←0

37:  **for all** cluster *C* with multiplicity m∈C  **do**

38:   score $\leftarrow 2\times m\times s_{T}(C,2\cdot m)$

39:   **if** score = 0 **then** score $\leftarrow m\times s_{T}(C,m)$

40:   totalScore ← totalScore + score

    **return** totalScore

### 3.2 Network construction

Given the consensus MUL-tree built by Algorithm 1, this section details how Polyphest converts it into a network that accounts for potential gene tree discordance caused by ILS. Polyphest builds on the algorithm originally proposed by Huber *et al.* (2006) and later implemented in PADRE, which identifies identical (isomorphic) clades within the MUL-tree, representing polyploidization events, and iteratively merges them. This approach minimizes the number of reticulation nodes. However, this strict requirement of clade isomorphism impacts inference negatively when gene tree discordance due to ILS is present.

To address this limitation, we introduce a near-isomorphism threshold (*δ*) to account for minor variations in clade topology. The improved algorithm operates directly on the input MUL-tree *T* and utilizes pre-computed cluster frequencies $s_{T}$ from the MUL-tree construction phase (Algorithm 1). The key steps are as follows:

1. Initialization
  - Assign isomorphism codes to all nodes within the MUL-tree for efficient comparison, following the approach described in Huber *et al.* (2006).
  - Initialize a list *L* of *H* + 1 queues. Each queue will store nodes at a specific height in the tree. The last queue L[H], will store the root node (at height *H*). All other queues, L[0] to L[H−1] will be initially empty.
2. Descending height processing
  - Starting from the maximum tree height (*H*), process nodes in descending order of height
    - For each queue L[h] (containing nodes at height *h*):
      - Use the AreNearIsomorphic function to identify nearly isomorphic clades within the threshold *δ*
      - Select the clade with the highest cumulative score as the representative clade
      - Use the MergeNearIsomorphicClades function to merge these identified clades into the representative clade
      - After processing a node, add its child nodes to their corresponding height queues (L[child height]) for future evaluation.
  - Repeat step 2 for all node heights, progressively refining the network by identifying and merging near-isomorphic clades until the MUL-tree is fully processed, resulting in the final network.

Polyphest differs from PADRE in two key aspects. First, it allows the merging of similar (not strictly identical) clades, which produces more accurate results in the presence of ILS. For example, while clades *T_u_* and *T_v_* shown in Fig. 1 are not isomorphic, by relaxing the isomorphism criterion with a threshold of 0.1, they could be considered potentially descended from a common ancestor through a polyploidization event. Second, Polyphest incorporates cluster frequencies among gene trees to select a representative clade during merging. This ensures the chosen representative reflects the most frequent cluster composition within the clade, leading to a network that more accurately reflects the underlying evolutionary history, especially when ILS is a factor.

## 4 Results

### 4.1 Simulation study

#### 4.1.1 Simulation setup

To evaluate the performance of Polyphest in simulations, we utilized the AlloppDT simulator (Jones 2012) to generate a collection of datasets. We replicated the scenarios explored in prior studies (Jones *et al.* 2013, Jones 2017), including varying numbers of allopolyploidization events (1, 2, or 3) and mutation rates to change the level of ILS. We substantially increased the number of genes to 1000, which not only reflects modern, genome-wide sequencing data but also allows us to evaluate the efficiency of Polyphest in handling large datasets, a known limitation of previous methods. We simulated gene sequences (500 bp, HKY model, transition/transversion ratio *κ *= 3 as in Jones 2017) for each dataset using Seq-Gen (Rambaut and Grassly 1997) and estimated gene trees from these alignments with IQ-TREE v2.1.2 (Minh *et al.* 2020). This procedure resulted in 720 simulated datasets (4 scenarios × 6 number of genes × 3 ILS levels × 10 replicates). Detailed descriptions of the model phylogenies (Supplementary Fig. S1) and the parameters used for simulations (Supplementary Table S1) are available in the Supplementary material. For comparison, we also ran PADRE and MPAllopp. PADRE was executed with a threshold parameter set to 2, indicating that only clusters appearing in at least two out of the input gene trees were considered when building the final consensus MUL-tree. Since the command-line version of PADRE only outputs a MUL-tree, we used the network construction algorithm in Polyphest (with an isomorphic threshold of 0 to match the original folding algorithm implemented in the GUI version of PADRE) to convert the output MUL-tree into a network for comparison. As a heuristic search-based method, MPAllopp, was run 30 times per dataset to improve the chance of finding the optimal solution. It was also supplied with the correct subgenome assignment.

#### 4.1.2 Evaluation metrics

To assess the accuracy of our MUL-tree and network constructions in the simulation study, we employed the normalized approximate graph edit distance implemented in NetworkX (Hagberg *et al.* 2008) to quantify the dissimilarity between the true MUL-tree and its estimated counterpart, with lower distance indicating higher accuracy. Similarly, we used the distance measure of Nakhleh (2010) to quantify the topological dissimilarity between the true and estimated networks. Details of the normalized graph edit distance and Nakhleh (2010) measure are provided in the Supplementary material (Section S2). We further evaluated computational efficiency by measuring the wall-clock time for each analysis (single-threaded), conducted on a Red Hat Enterprise Linux system with AMD EPYC 7642 48-Core Processor.

#### 4.1.3 Data characteristics

We measured the data complexity (incongruence) in three aspects: (i) the level of ILS, quantified by the normalized rooted RF (Robinson and Foulds 1981) distance between each pair of true species MUL-tree and the true gene tree; (ii) gene tree estimation error (GTEE), capturing the error introduced during individual gene tree reconstruction; (iii) average discordance, combining the discordance caused by ILS and GTEE, representing the overall incongruence between the true species MUL-tree and the estimated gene trees. The results are shown in Supplementary Table S2. We observe an increasing trend of discordance (ILS and average discordance) with higher mutation rates, attributable to decreased branch lengths (measured in units of generations) where ILS is more prevalent. This suggests that higher mutation rates make reconstructing the true species phylogeny more challenging. GTEE generally decreases with higher mutation rates, except for network J. This pattern might be explained by saturation effects in network J, where high mutation rates on already long branches (representing more generations of change) have a diminishing impact on gene tree inference accuracy.

**Figure 2.**
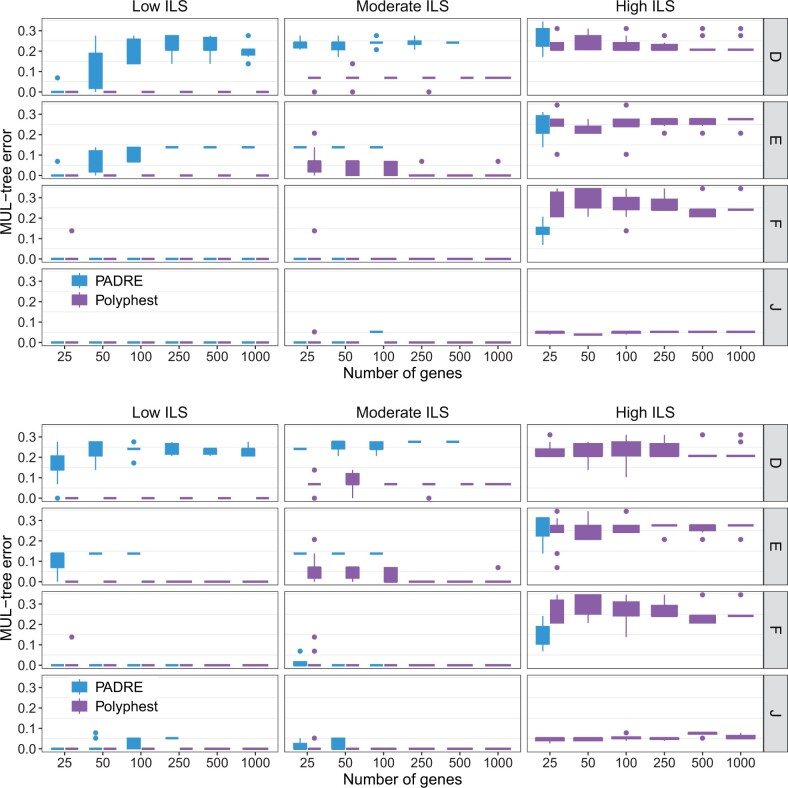
MUL-tree reconstruction error. The normalized approximate graph edit distance between the true MUL-tree and the MUL-tree reconstructed from true gene trees (top) and from estimated gene trees (bottom). Results are shown for 10 replicate datasets

**Figure 3.**
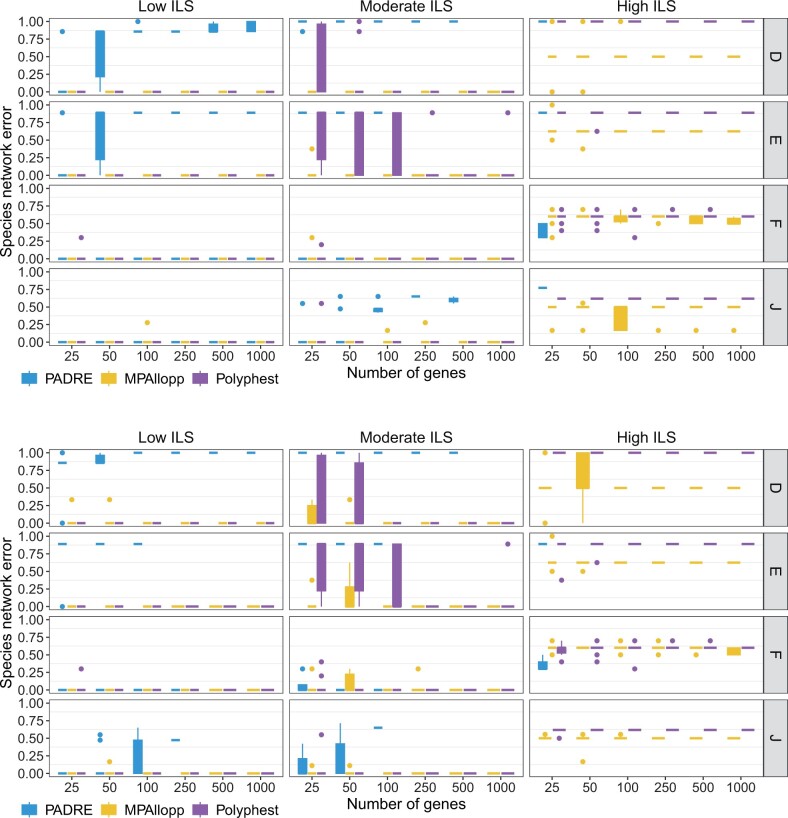
Species network estimation error. The normalized network distance between the true network and the network reconstructed from true gene trees (top) and from estimated gene trees (bottom). Results are shown for 10 replicate datasets

It is important to note that ILS could be problematic for PADRA. Indeed, PADRE failed on 20 out of 480 low ILS datasets, 294 out of 480 moderate ILS datasets, and 454 out of 480 high ILS datasets across different scenarios and data types (true and estimated gene trees). These failures are excluded from the results discussed next with respect to Figs 2 and 3.

#### 4.1.4 Accuracy of MUL-tree reconstruction

We evaluated the performance of the MUL-tree reconstruction algorithms employed by PADRE and by Polyphest (Fig. 2). MPAllopp does not reconstruct a MUL-tree. As the level of ILS increases, we observe a significant decline in PADRE’s performance. The failure rate of PADRE in generating MUL-tree rose sharply from 4.17% at low ILS levels to an alarmingly high 94.6% at a high level of ILS. Notably, under high ILS conditions, PADRE failed to produce any results, with successful cases limited to scenarios involving a small number of genes (high ILS with 25 genes). In contrast, Polyphest consistently outperformed PADRE. It exhibits perfect accuracy under low ILS levels and remains highly accurate under moderate ILS levels. Additionally, its performance improved as the number of genes increased. Unsurprisingly, for very high ILS levels, the method’s performance gets poorer, but it still achieves an accuracy level above 70%. Furthermore, gene tree error has a negligible impact on the performance of Polyphest, whereas its impact on PADRE is more noticeable.

#### 4.1.5 Accuracy of network reconstruction

We evaluated the accuracy of the networks reconstructed by PADRE, MPAllopp, and Polyphest (Fig. 3). While PADRE shows promise for low ILS conditions and low numbers of genes, its inability to handle a significant portion of the data, especially under high ILS and with more genes, limits its overall reliability and applicability to complex evolutionary scenarios. In contrast, MPAllopp and Polyphest have very high accuracy across all scenarios under low ILS levels, and comparable, yet lower, accuracy on the species networks of scenarios F and J for high levels of ILS (bottom two rows in Fig. 3). MPAllopp has better accuracy for moderate and high levels of ILS on the species networks of scenarios D and E. Furthermore, for moderate levels of ILS, increasing the number of genes leads to a drastic improvement in the performance of Polyphest, especially on the species networks of scenarios D and E.

**Figure 4.**
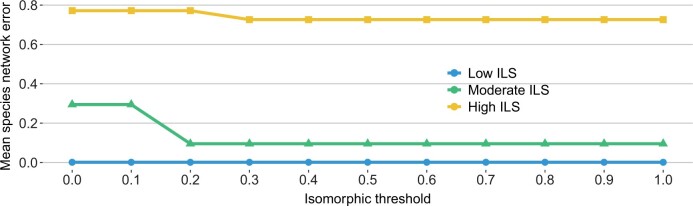
Impact of isomorphic threshold on species network error. Results are shown across various scenarios considering different numbers of genes and data types (true and estimated gene trees)

We further explored the impact of the isomorphic threshold (*δ* above) on the accuracy of network reconstruction by Polyphest. This threshold is designed to handle ILS; therefore, we expect the level of ILS to play a role in the method’s performance for the same setting of the threshold value. Indeed, as Fig. 4 shows, for the same setting of a threshold value, Polyphest is most accurate on low ILS and least accurate on high ILS. An encouraging result is that a threshold value of 0.3 or higher produces the most accurate results under all levels of ILS, and varying its value beyond 0.3 barely has an impact. Furthermore, for low levels of ILS, very low threshold values would also work, as the strict requirement of isomorphism in those cases is not detrimental. Nevertheless, given the stability in performance for threshold values higher than 0.3, it seems a safer choice to use such values in the analyses.

#### 4.1.6 Runtime

**Figure 5.**
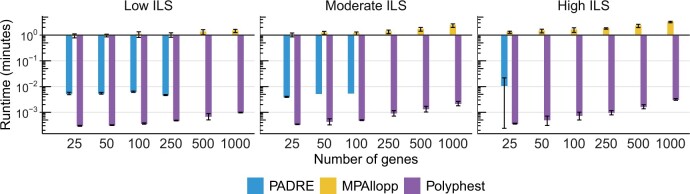
Average running time (minutes) measured by wall-clock time on simulated data under scenario F (5-taxon with three reticulations). Error bars represent the standard deviation. Inferences were performed on estimated gene trees

We evaluated the runtime performance of each method by recording their wall-clock time to complete a single analysis on a single thread. It is important to note that MPAllopp utilizes a heuristic search, requiring multiple runs to improve its search outcome. Therefore, the reported runtime in Fig. 5 reflects the average time required for 30 such runs. Polyphest is the fastest method, consistently finishing within 0.15 seconds for the 5-taxon dataset with 1000 genes and averaging around 0.4 seconds for the 13-taxon dataset with 1000 genes. PADRE generally completed analysis within 10 seconds, including the time required for MUL-tree folding. However, it is important to note that PADRE failed on 37 out of 60 low ILS datasets, 54 out of 60 moderate ILS datasets, and 57 out of 60 high ILS datasets in scenario F. These failures are excluded from the results presented in Fig. 5. MPAllopp had the slowest runtime and the numbers of reticulations and taxa significantly impacted its performance. For the 5-taxon dataset with 1000 genes, the average runtime for MPAllopp (averaged over 30 runs) increased from 0.226 seconds with one reticulation (scenario D) to 1.35 seconds with two reticulations (scenario E) and further to 4.36 seconds with three reticulations (scenario F). This trend continued for the 13-taxon dataset with three reticulations (scenario J), where MPAllopp typically required approximately 24.7 minutes to finish a single search round (Supplementary Fig. S2).

### 4.2 Empirical data analysis

**Figure 6.**
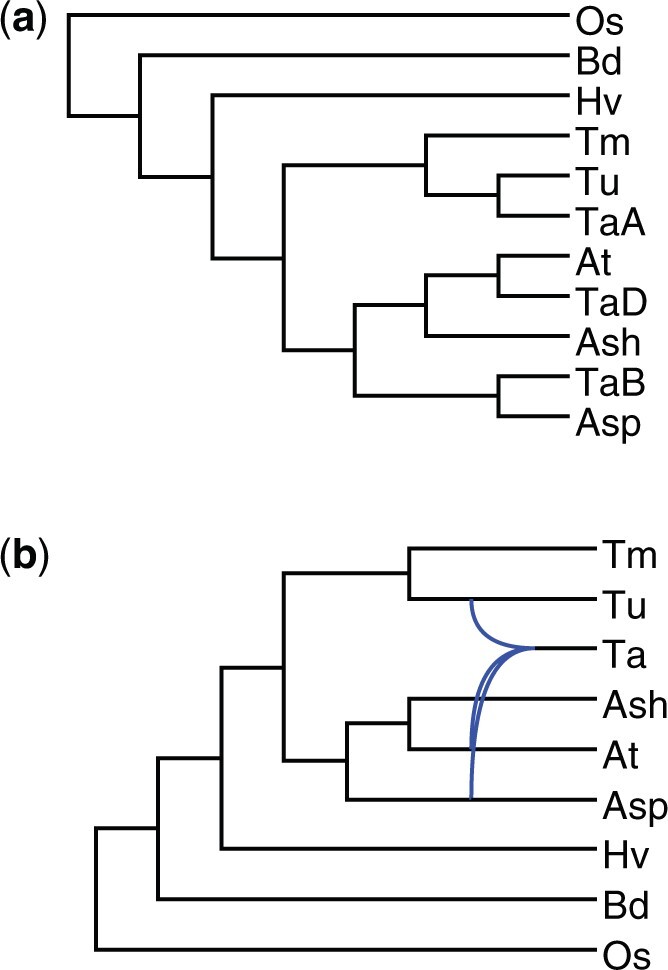
Bread wheat phylogenies. The taxa are “TaA” (*Triticum aestivum A subgenome*), “TaB” (*T. aestivum B subgenome*), “TaD” (*T. aestivum D subgenome*), “Tu” (*T. urartu*), “Tm” (*T. monococcum*), “Ash” (*Aegilops sharonensis*), “Asp” (*Ae. speltoides*), “At” (*Ae. tauschii*), “Hv” (*Hordeum vulgare*), “Bd” (*Brachypodium distachyon*), and “Os” (*Oryza sativa*)

We re-analyzed the 9-species bread wheat data from Marcussen *et al.* (2014) which includes one hexaploid bread wheat (*Triticum aestivum*), five diploid relatives, and three outgroup species. This dataset comprises 275 genome-wide collection of gene trees. To gain insights into the subgenome relationships, we first performed an analysis considering the A, B, and D subgenomes of the hexaploid bread wheat (denoted as TaA, TaB, and TaD, respectively) as distinct taxa. This approach produced a MUL-tree showing the specific subgenome and its closest relative (Fig. 6a). We then conducted an analysis without distinguishing between the A, B, and D subgenomes, treating the input gene trees as MUL-trees. This resulted in a network that captures the overall evolutionary history of the bread wheat data (Fig. 6b). For these analyses, we used a filtering threshold of 21, and an isomorphic threshold of 0.2.

The MUL-tree inferred by Polyphest is consistent with the underlying MUL-tree reported in Yan *et al.* (2021) using MPAllopp. In addition, Polyphest identified the same diploid progenitors for each subgenome as Marcussen *et al.* (2014): subgenome TaA is sister to diploid *Triticum urartu*, subgenome TaB is sister to diploid *Aegilops speltoides*, and subgenome TaD is sister to diploid *Aegilops tauschii* (Fig. 6a). Notably, the network reconstructed by Polyphest achieves consensus with the two optimal solutions identified by MPAllopp, both of which involve two hybridization events.

## 5 Discussion

In this work, we introduced Polyphest, a novel method that directly infers species phylogenies from gene trees while accounting for polyploidy and ILS. Polyphest extends the PADRE algorithm by accounting for ILS. Unsurprisingly, Polyphest exhibits greater robustness to ILS compared to PADRE. Further, Polyphest demonstrates comparable performance to MPAllopp under low to moderate ILS conditions, particularly with hundreds of genes. However, Polyphest offers a significant advantage in computational efficiency, especially for large datasets, which makes it more scalable than other methods. In reanalyzing a bread wheat dataset, Polyphest inferred a plausible phylogeny, corroborating previous research findings, and provided insights into the origin of subgenomes within the hexaploid bread wheat, showcasing its practical utility.

While Polyphest performs well under low and moderate ILS conditions, its performance suffers under high ILS levels, leading to an overestimation of the number of reticulations (polyploidization events). This limitation arises because, under high ILS, the most frequent gene relationships might not reflect true species relationships. Additionally, with an insufficient number of genes, all possible relationships might appear in the gene trees with similarly low frequencies, resulting in substantial uncertainty. Improving the selection of near-isomorphic clades during network reconstruction could improve the method’s performance under high levels of ILS, and we identify this as a direction for future research.

## Supplementary Material

btae390_Supplementary_Data

## Data Availability

The data underlying this article are available in the article and in its online supplementary material.
