# Supplementary material for: Polyphest: fast polyploid phylogeny estimation
Source: Bioinformatics. 2024 Sep 4;40(Suppl 2):ii20–8. doi: 10.1093/bioinformatics/btae390 (PMC11373313; doi:10.1093/bioinformatics/btae390)
Supplement: btae390_Supplementary_Data [file btae390_supplementary_data.zip › PolyphestSupplementary.pdf]

Polyphest: Fast Polyploid Phylogeny Estimation

Supplementary Material

Zhi Yan[1,*], Zhen Cao[1], and Luay Nakhleh[1,2,*]

[1]Department of Computer Science, Rice University, Houston TX, 77005, USA

[2]Department of BioSciences, Rice University, Houston TX, 77005, USA

[*]zhi.yan@rice.edu, nakhleh@rice.edu

Contents

S1 Simulation settings **S2**

S2 Evaluation **S3**

S2.1 MUL-tree distance S3

S2.2 Network distance S4

S3 Results on simulated data **S4**

S3.1 Data characteristics S4

S4 Commands **S6**

S1 Simulation settings

We used the phylogenetic networks from Jones *et al.* (2013); Jones (2017) as our model phylogenies. Since all evaluated methods required rooted gene trees, we added an outgroup to each model phylogeny by extending the root's stem branch to a length of 7 coalescent units.

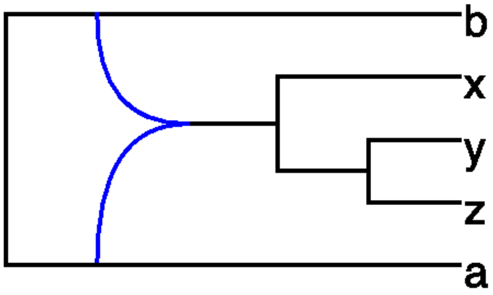

(a) Scenario D

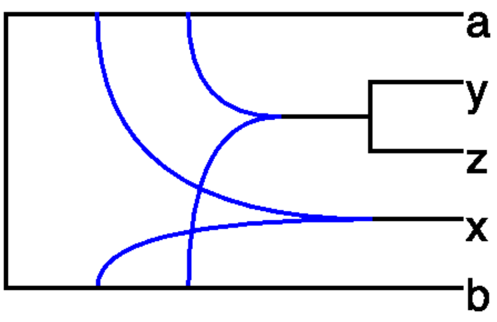

(b) Scenario E

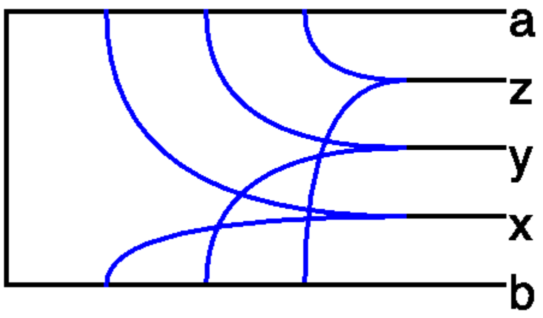

(c) Scenario F

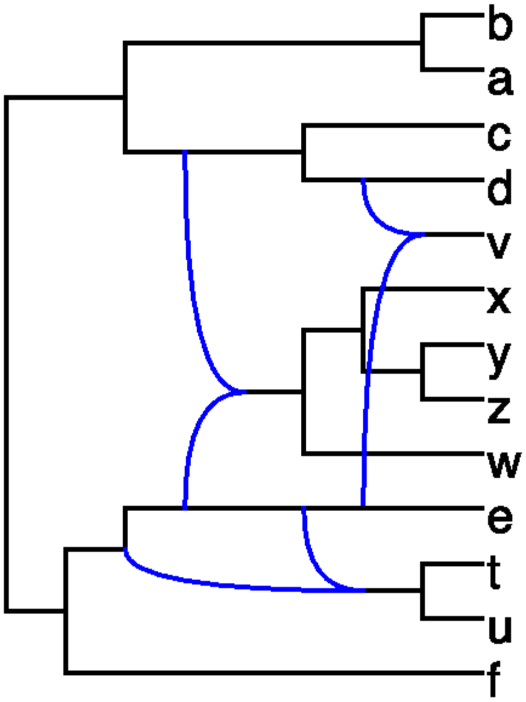

(d) Scenario J

Figure S1: Model phylogenies obtained from Jones *et al.* (2013); Jones (2017).

Table S1: **AlloppDT parameters for simulated data.** We simulated 72 conditions by varying AloppDT parameters based on the species phylogenies illustrated in Figure S1. For each condition, 10 replicate simulations were performed.

Scenario	Parameters
D E F	G (Number of genes) $\in \{25, 50, 100, 250, 500, 1000\}$<br>N (Individuals per species) = 1<br>T (Mutation rates) $\in \{4e-9, 2e-8, 1e-7\}$ substitutions per site per generation<br>H (Ingroup root height)= 0.012 substitutions per site
J	G (Number of genes) $\in \{25, 50, 100, 250, 500, 1000\}$<br>N (Individuals per species) = 1<br>T (Mutation rates) $\in \{4e-9, 2e-8, 1e-7\}$ substitutions per site per generation<br>H (Ingroup root height)= 0.035 substitutions per site

S2 Evaluation

S2.1 MUL-tree distance

To evaluate the accuracy of MUL-tree construction, we used an approximate version of the graph edit distance. This metric, implemented by the `networkx.optimize_graph_edit_distance(T1,T2)` function (Hagberg *et al.* (2008)), calculates the minimum number of edits operations required to transform one MUL-tree ($T1$) into another ($T2$). We opted for the approximate approach due to the computational cost of the exact graph edit distance, known to be NP-hard. Additionally, we normalized the distance by dividing it with the upper bound based on the number of nodes and edges in the trees. The specific calculation for the normalized distance is shown in the code snippet below.

```
distance = networkx.optimize_graph_edit_distance(
    T1, T2, node_match=lambda u, v: u["species"] == v["species"]
)


normalization = max(len(T1.nodes) + len(T1.edges), len(T2.nodes) + len(T2.edges))
normalized_distance = distance / normalization
```

S2.2 Network distance

The accuracy of the phylogenetic network estimates was assessed using the Nakhleh distance (Nakhleh (2010)). This metric evaluates the distance between two networks, $\psi = (V, E)$ and $\hat{\psi} = (\hat{V}, \hat{E})$, as follows:

$$d(\psi, \hat{\psi}) = 0.5(\sum_{v \in U(\psi)} max\{0, \kappa(v) - \kappa(v')\} + \sum_{u \in U(\hat{\psi})} max\{0, \kappa(u) - \kappa(u')\}),$$

where v' represents a node in $\hat{\psi}$ that is equivalent to v in ψ, and similarly, u' is equivalent to u in ψ. Nodes v and v' are considered equivalent, denoted $v \equiv v'$, if they are either leaves with the same label or if all their children are equivalent. $\kappa(v)$ indicates the count of nodes equivalent to a given node v within ψ. The set $U(\psi)$ denotes the unique nodes in ψ that do not have an equivalent in $\hat{\psi}$.
In the scenario where the network inferred, $\hat{\psi}$, lacks any nodes equivalent to those in the true network ψ, the maximal distance between ψ and $\hat{\psi}$ can be represented as the average number of internal nodes in both ψ and $\hat{\psi}$, calculated as $n - 1 + r + \hat{r}$, where n represents the total number of leaves, while r and $\hat{r}$ represent the counts of reticulation nodes in the true and inferred networks, respectively.

S3 Results on simulated data

S3.1 Data characteristics

Table S2: **Characteristics of simulated data.** The levels of ILS, gene tree estimation error (GTEE), and averge discordance (AD) of the various simulated data sets. The species network labels refer to four scenarios shown in Fig. S1. Each reported value indicates the mean ± standard deviation based on 10 replicates for each model condition.

Species network	Mutation rate (per site per generation)	ILS	GTEE	AD
D	4×10^{-9}	0.046 ± 0.002	0.276 ± 0.003	0.291 ± 0.003
	2×10^{-8}	0.488 ± 0.005	0.251 ± 0.005	0.560 ± 0.005
	1×10^{-7}	0.841 ± 0.003	0.125 ± 0.004	0.847 ± 0.003
E	4×10^{-9}	0.047 ± 0.002	0.272 ± 0.004	0.287 ± 0.004
	2×10^{-8}	0.496 ± 0.006	0.249 ± 0.004	0.566 ± 0.005
	1×10^{-7}	0.842 ± 0.004	0.125 ± 0.003	0.848 ± 0.003
F	4×10^{-9}	0.051 ± 0.002	0.279 ± 0.004	0.296 ± 0.003
	2×10^{-8}	0.507 ± 0.004	0.250 ± 0.004	0.576 ± 0.004
	1×10^{-7}	0.847 ± 0.005	0.124 ± 0.003	0.852 ± 0.005
J	4×10^{-9}	0.002 ± 0.0003	0.105 ± 0.002	0.106 ± 0.002
	2×10^{-8}	0.130 ± 0.002	0.130 ± 0.002	0.201 ± 0.002
	1×10^{-7}	0.578 ± 0.002	0.138 ± 0.002	0.595 ± 0.002

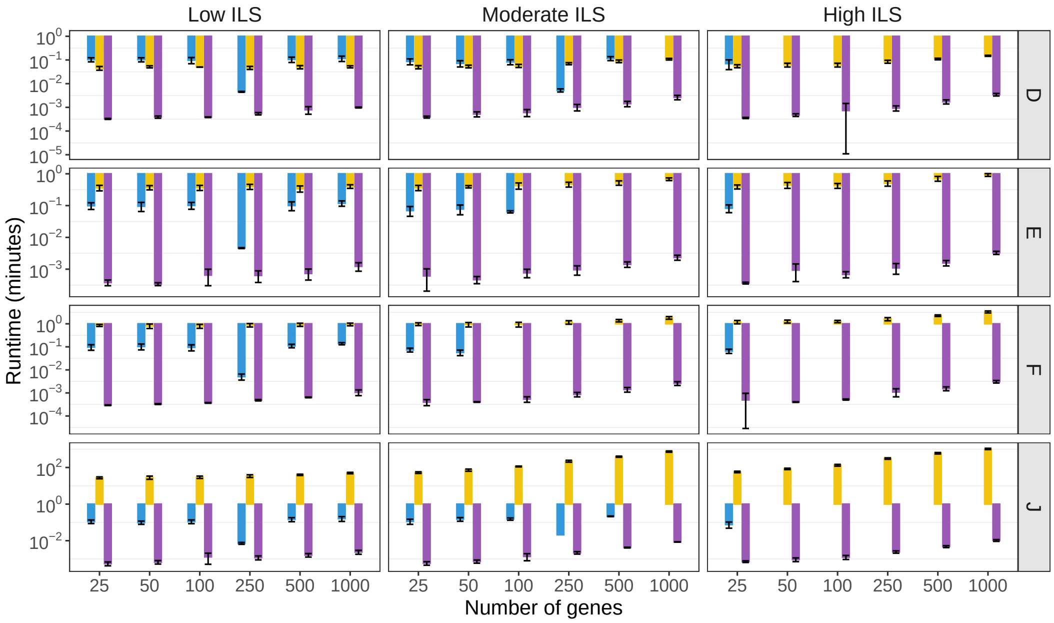

(a) True gene trees

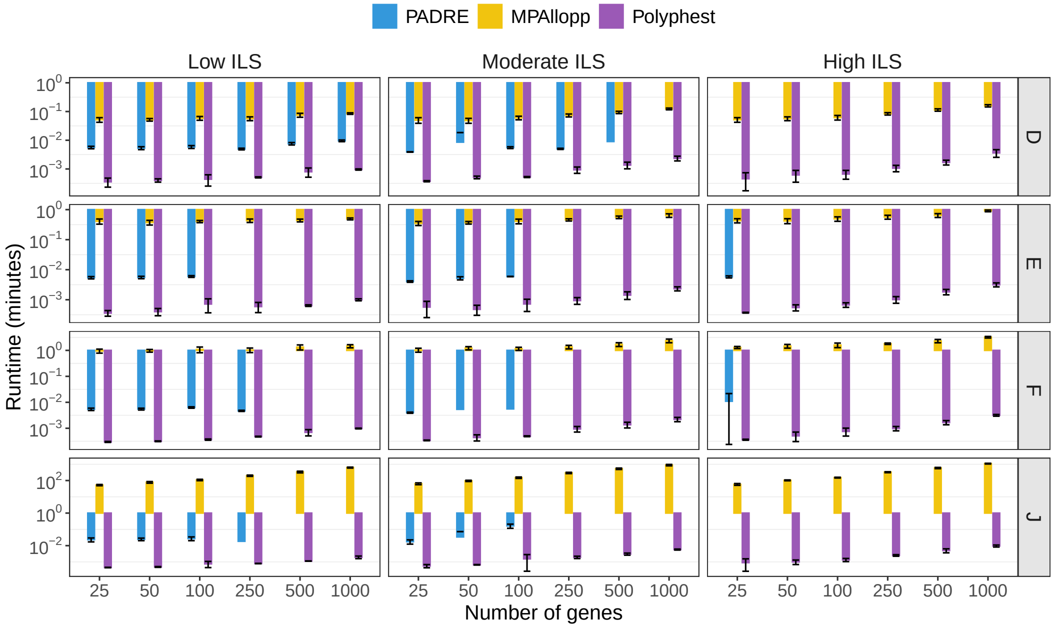

(b) Estimated gene trees

Figure S2: Average running time (minutes) measured by wall-clock time on simulated data. Top: Inferences were performed on true gene trees. Bottom: Inferences were performed on estimated gene trees. Error bars represent the standard deviation. The y-axis is on a logarithmic scale.

S4 Commands

IQ-TREE v2.1.3 (Minh *et al.* (2020)) was run as

```
iqtree2 -s <sequence file> -o 01oA
```

PADRE (Lott *et al.* (2009)) was run as

```
java -jar padre-cli.jar -i <gene tree file> -t <threshold=2> -o b
```

References

Hagberg, A. *et al.* (2008). Exploring network structure, dynamics, and function using networkx. Technical report, Los Alamos National Lab.(LANL), Los Alamos, NM (United States).

Jones, G. (2017). Bayesian phylogenetic analysis for diploid and allotetraploid species networks. *bioRxiv*.

Jones, G. *et al.* (2013). Statistical inference of allopolyploid species networks in the presence of incomplete lineage sorting. *Systematic Biology*, **62**(3), 467–478.

Lott, M. *et al.* (2009). Inferring polyploid phylogenies from multiply-labeled gene trees. *BMC evolutionary biology*, **9**(1), 1–11.

Minh, B. Q. *et al.* (2020). IQ-TREE 2: new models and efficient methods for phylogenetic inference in the genomic era. *Molecular Biology and Evolution*, **37**(5), 1530–1534.

Nakhleh, L. (2010). A metric on the space of reduced phylogenetic networks. *IEEE/ACM Transactions on Computational Biology and Bioinformatics (TCBB)*, **7**(2), 218–222.
