# Supplementary material for: Polyphest: fast polyploid phylogeny estimation
Source: Bioinformatics. 2024 Sep 4;40(Suppl 2):ii20–8. doi: 10.1093/bioinformatics/btae390 (PMC11373313; doi:10.1093/bioinformatics/btae390)
Supplement: btae390_Supplementary_Data [file btae390_supplementary_data.zip › PolyphestResponseLetter.docx]

*We thank the reviewers for their positive feedback and the ECCB2024 organising committee for considering our paper titled “Polyphest: Fast Polyploid Phylogeny Estimation” for conditional acceptance in ECCB proceedings. We have addressed all reviewer comments in the revised manuscript, and provide our responses below.*

Review 1

Review comments

The authors present a novel algorithm for inferring phylogenetic trees while accounting for polyploidy and incomplete lineage sorting by first clustering the input gene trees and then introducing a near-isomorphism threshold (as compared to strictly isomorphic clades), thereby improving performance regarding both accuracy and processing time when compared to two existing tools. The manuscript is well written and background, motivation and methodology are comprehensively and understandably explained, which makes the study suitable for a broader audience. I recommend accepting the mauscript with minor corrections.

*We thank the reviewer for their encouraging comments.*

1. The sentence "First, it allows merging of similar (not strictly identical) clades, which produces more accurate results in the presence of ILS might." in the Network Construction section on page 4 is not clear. Please check and potentially correct it.

*Thank you for pointing this out. It has now been corrected.*

2. Please correct the sentence "Polyphest Polyphest extends the PADRE algorithm by accounting for ILS." in the Discussion section on page 7.

*It has now been corrected as well.*

Review 2

Review comments

New, improved algorithm for polyploid phylogeny, also takes into account incomplete lineage sorting. Well written. Introduces clever relaxed notions making the problem formulation more realistic. Gives ILP solution for hard problem. Good results.

*We appreciate the reviewer’s positive response to our work.*
